# Optimization of wavefront reconstruction accuracy for conjugate shift differential absolute testing

**DOI:** 10.1038/s41598-022-26380-y

**Published:** 2022-12-16

**Authors:** Xueliang Zhu, Dasen Wang, Mengyao Zhang, Bingcai Liu, Ailing Tian, Guiying Jin, Xianfeng Zheng

**Affiliations:** 1 School of Optoelectronic Engineering, Xi’an Technological University, Xi’an, China; 2 Advanced Manufacturing Institute, Inner Mongolia Institute of Metal Materials, Ningbo, China

**Keywords:** Engineering, Optics and photonics

## Abstract

The conjugate shift differential method, based on Fourier transforms, is critical for surface error testing of high-precision optical elements. However, this common approach is also prone to periodic spectrum loss. As such, this paper proposes conjugate double shift differential (CDSD) absolute testing, which can effectively compensate for spectrum loss and achieve accurate wavefront reconstructions. Spectrum loss in the single shift differential method is analyzed through a study of the Fourier reconstruction process. A calculation model for the proposed CDSD method is then established and constraint conditions for shift quantities are provided by analyzing double shear effects observed in transverse shear interference. Finally, the reconstruction accuracies of various spectrum compensation methods are compared. Results showed that spectrum loss became more evident with increasing shift amounts. However, the CDSD method produced the smallest measurement error compared with conventional direct zero filling and adjacent point averaging, suggesting our approach could effectively improve absolute shape measurement accuracy for planar optical elements.

## Introduction

High-precision measurements are a prerequisite for precision machining. Specifically, systems utilizing inertial confinement fusion^1^ or extreme ultraviolet lithography^2,3^ include stringent requirements for the surface shape accuracy of optical elements^4^. Absolute testing technology based on traditional interferometry^5–7^ improves mirror shape measurement accuracy by eliminating the influence of reference mirror shape errors. As such, the absolute testing principle for conjugate shift differentials^8^ is similar to that of transverse shear interferometry, as both calculate the absolute shape of a measured surface after reconstructing the differential shape. However, the use of a Fourier method for reconstruction leads to missing spectrum information at coordinate points that are integer multiples of n/s, which causes uncertainty in some wavefront coefficients and affects surface shape reconstruction accuracy^9^. To solve this problem, spectrum compensation methods are applied in transverse shearing interferometry, including the direct zero compensation, adjacent point averaging, interpolation, and double shearing methods.

Uncertain points can be processed by zero filling, but the resulting error is large^10^. In 2000, Elster et al.^11^ proposed a double shear scheme to compensate for a missing spectrum in the reconstruction of transverse shear interference wavefronts. In this process, two groups of differential phases, corresponding to two shear quantities without common factors, were calculated and used to determine exact expansion coefficients. Measured wavefronts were then reconstructed using an inverse Fourier transform of these coefficients. In 2006, Liang et al.^12^ used the average values of adjacent points to interpolate a missing frequency spectrum. This approach is applicable to relatively continuous and gentle wavefront reconstructions in which the shear amount is no more than 1/8 the diameter of the measured mirror (high reconstruction error). In 2007, Claas et al.^13^ proposed a spectrum compensation method based on Shannon interpolation, in which a sinc-based function was inserted into the Fourier coefficient points for each differential phase, then the points that could not be determined in the spectrum were avoided during resampling, it is suitable for wavefront reconstruction of abrupt phase. In 2012, Guo et al.^14^ proposed a multi-shear interferometry technique that effectively solved the problem of spectrum loss and improved the signal-to-noise ratio by using multiple groups of interference data. The resulting reconstruction accuracy improved with an increasing number of shear quantities. In the present study, a conjugate double shift differential (CDSD) method is proposed, based on double shearing in transverse shearing interferometry, which can accurately reconstruct wavefronts and solve the problem of missing spectra in the absolute testing of conjugate shift differentials. A series of experiments were conducted to validate the proposed methodology, as discussed in the following sections.

## Spectrum compensation in the conjugate double shift differential method

**Figure 1:**
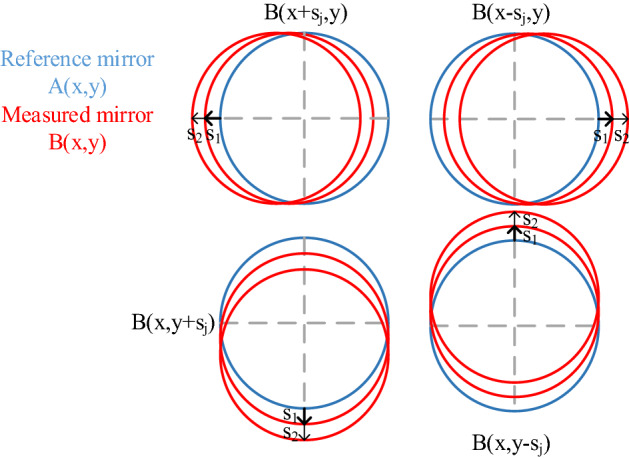
The proposed conjugate double shift differential absolute testing principle.

The conjugate double shift differential (CDSD) method involves adding interferometry data on the basis of the original conjugate single shift differential (CSSD) method^8^. During this test, the position of a reference mirror is held fixed, and the test mirror is conjugate translated a distance s_j_ along the orthogonal direction of the reference mirror, as shown in Fig. 1. The term *A(x,y)* denotes the shape profile of the reference mirror, *B(x,y)* is the measured mirror shape, and *W(x,y)* is the measured interference wave surface. The eight groups of measurement results can be represented as:

1 $$ \left\{ {\begin{array}{*{20}c} {\begin{array}{*{20}c} {\begin{array}{*{20}c} {W_{1}^{j} (x,y){ = }A(x,y) + B(x + s_{j} ,y)} \\ {W_{2}^{j} (x,y){ = }A(x,y) + B(x - s_{j} ,y)} \\ \end{array} } \\ {W_{3}^{j} (x,y){ = }A(x,y) + B(x,y + s_{j} )} \\ \end{array} } \\ {W_{4}^{j} (x,y){ = }A(x,y) + B(x,y - s_{j} )} \\ \end{array} } \right.\;j = 1,2 $$

The differential surfaces $\Delta W_{x}^{j} (x,y)$ and $\Delta W_{y}^{j} (x,y)$ on the tested mirror can then be acquired in two orthogonal directions by processing data at conjugate positions in the same direction on the tested mirror. Shape errors for the reference mirror can be eliminated as follows:

2 $$ \left\{ {\begin{array}{*{20}c} {\Delta W_{x}^{j} (x,y) = \frac{{W_{1}^{j} (x,y) - W_{2}^{j} (x,y)}}{{2s_{j} }} = \frac{{B(x + s_{j} ,y) - B(x - s_{j} ,y)}}{{2s_{j} }}} \\ {\Delta W_{y}^{j} (x,y) = \frac{{W_{3}^{j} (x,y) - W_{4}^{j} (x,y)}}{{2s_{j} }} = \frac{{B(x,y + s_{j} ) - B(x,y - s_{j} )}}{{2s_{j} }}} \\ \end{array} } \right. $$

The absolute surface shape of the mirror being tested can be obtained from wavefront reconstruction calculations based on a Fourier transform of the differential surface shape represented by Eq. (2). The measured wavefront can be described by a Fourier series and the Fourier transform coefficients of the measured wavefront can be obtained from the differential wavefront. Wavefront information can then be reconstructed using an inverse Fourier transform of the resulting coefficients^15,16^.

The Fourier series expansion of the measured mirror shape is given by:

3 $$ W(x,y) = \sum\limits_{p = 0}^{N - 1} {\sum\limits_{q = 0}^{N - 1} {a_{pq} Z_{pq} } } (x,y)\;x,y = 0,1,2,...N - 1, $$

4 $$ Z_{pq} (x,y) = \frac{1}{N}\exp \left[ {\frac{2\pi i}{N}\left( {px + qy} \right)} \right]\;p,q = 0,1,2,...N - 1, $$

where *α* is the expansion coefficient and *p* and *q* are the corresponding frequency domain coordinates. The following relationship is evident from Eq. (3):

5 $$ W(x,y) = FT_{pq}^{ - 1} \left\{ {a_{pq} } \right\} $$

Substituting Eq. (3) into (2) allows the differential shape of the measured surface in the x direction to be expressed as:

6 $$ \begin{gathered} \Delta W_{x}^{j} (x,y) = \left\{ {\sum\limits_{p = 0}^{N - 1} {\sum\limits_{q = 0}^{N - 1} {a_{{_{pq} }}^{j} Z_{pq} } } (x + s_{j} ,y) - \sum\limits_{p = 0}^{N - 1} {\sum\limits_{q = 0}^{N - 1} {a_{{_{pq} }}^{j} Z_{pq} } } (x - s_{j} ,y)} \right\}/2s_{i} \hfill \\ = \sum\limits_{p = 0}^{N - 1} {\sum\limits_{q = 0}^{N - 1} {a_{{_{pq} }}^{j} Z_{pq} } } (x,y)\left[ {\exp \frac{{ip\pi 2s_{j} }}{N} - \exp ( - \frac{{ip\pi 2s_{j} }}{N})} \right]/2s_{j} \hfill \\ = \sum\limits_{p = 0}^{N - 1} {\sum\limits_{q = 0}^{N - 1} {a_{{_{pq} }}^{j} Z_{pq} } } (x,y)\left[ {2i\sin \left( {\frac{{p\pi 2s_{j} }}{N}} \right)} \right]/2s_{j} \hfill \\ \end{gathered} $$

Similarly, the differential shape of the measured surface in the y direction can be represented by:

7 $$ \begin{gathered} \Delta W_{y}^{j} (x,y) = \left\{ {\sum\limits_{p = 0}^{N - 1} {\sum\limits_{q = 0}^{N - 1} {a_{{_{pq} }}^{j} Z_{pq} } } (x,y + s_{j} ) - \sum\limits_{p = 0}^{N - 1} {\sum\limits_{q = 0}^{N - 1} {a_{{_{pq} }}^{j} Z_{pq} } } (x,y - s_{j} )} \right\}/2s_{j} \hfill \\ = \sum\limits_{p = 0}^{N - 1} {\sum\limits_{q = 0}^{N - 1} {a_{{_{pq} }}^{j} Z_{pq} } } (x,y)\left[ {\exp \frac{{iq\pi 2s_{j} }}{N} - \exp ( - \frac{{iq\pi 2s_{j} }}{N})} \right]/2s_{j} \hfill \\ = \sum\limits_{p = 0}^{N - 1} {\sum\limits_{q = 0}^{N - 1} {a_{{_{pq} }}^{j} Z_{pq} } } (x,y)\left[ {2i\sin \left( {\frac{{q\pi 2s_{j} }}{N}} \right)} \right]/2s_{j} \hfill \\ \end{gathered} $$

Fourier coefficients for the measured surface can be acquired using a least-squares method:

8 $$ a_{{_{pq} }}^{j} = \frac{1}{{2i\left[ {\sin^{2} \left( {\frac{{p\pi 2s_{j} }}{N}} \right) + \sin^{2} \left( {\frac{{q\pi 2s_{j} }}{N}} \right)} \right]}}\left\{ {\sin \left( {\frac{{p\pi 2s_{j} }}{N}} \right) \times FT\left[ {\Delta W_{x}^{j} (x,y)} \right] + \sin \left( {\frac{{q\pi 2s_{j} }}{N}} \right) \times FT\left[ {\Delta W_{y}^{j} (x,y)} \right]} \right\} $$

It is evident from Eq. (8) that in the case of only one group of interference results (i.e., the value of *j* is 1) the denominator of the wavefront coefficient is zero, since *α*_pq_ = *p* and *q* = *KN/2 s* (*k* is a positive integer). In other words, part of the spectrum is missing, which increases surface reconstruction errors. Therefore, accurately reconstructing measured surface shapes requires determining all of the spectral coefficients *α*_pq_. As a result, at least two shift quantities must be used to achieve relative measurements since the shift quantities s_1_ and s_2_ have no common factors. These shift constraints are given by:

9 $$ \left\{ {\begin{array}{*{20}c} {\begin{array}{*{20}c} {GCD(s_{1} ,s_{2} ) = 1} \\ {s_{1} + s_{2} \le N} \\ \end{array} } \\ {s_{1} ,s_{2} > 1} \\ \end{array} } \right. $$

where GCD is the maximum common divisor and N is the number of sampling points. When the p and q values in Eq. (8) are equal to *KN/2s*_1_, the denominator term is 0. However, the denominator corresponding to s_2_ is not 0, due to the coprime relationship in the shift amount. The spectrum coefficient loss for a shift amount of s_1_ can be compensated for using the spectrum coefficient for a shift amount of s_2_. In other words, the spectrum coefficient loss for a larger shift can be compensated for using the spectrum coefficient for a smaller shift. In addition, all spectral coefficients (excluding *α*_00_) can be determined using two shift quantities without common factors. The coefficient *α*_00_ only affects the offset of a reconstructed surface and not the surface accuracy.

## Simulations and analysis of surface reconstruction accuracy

**Figure 2:**
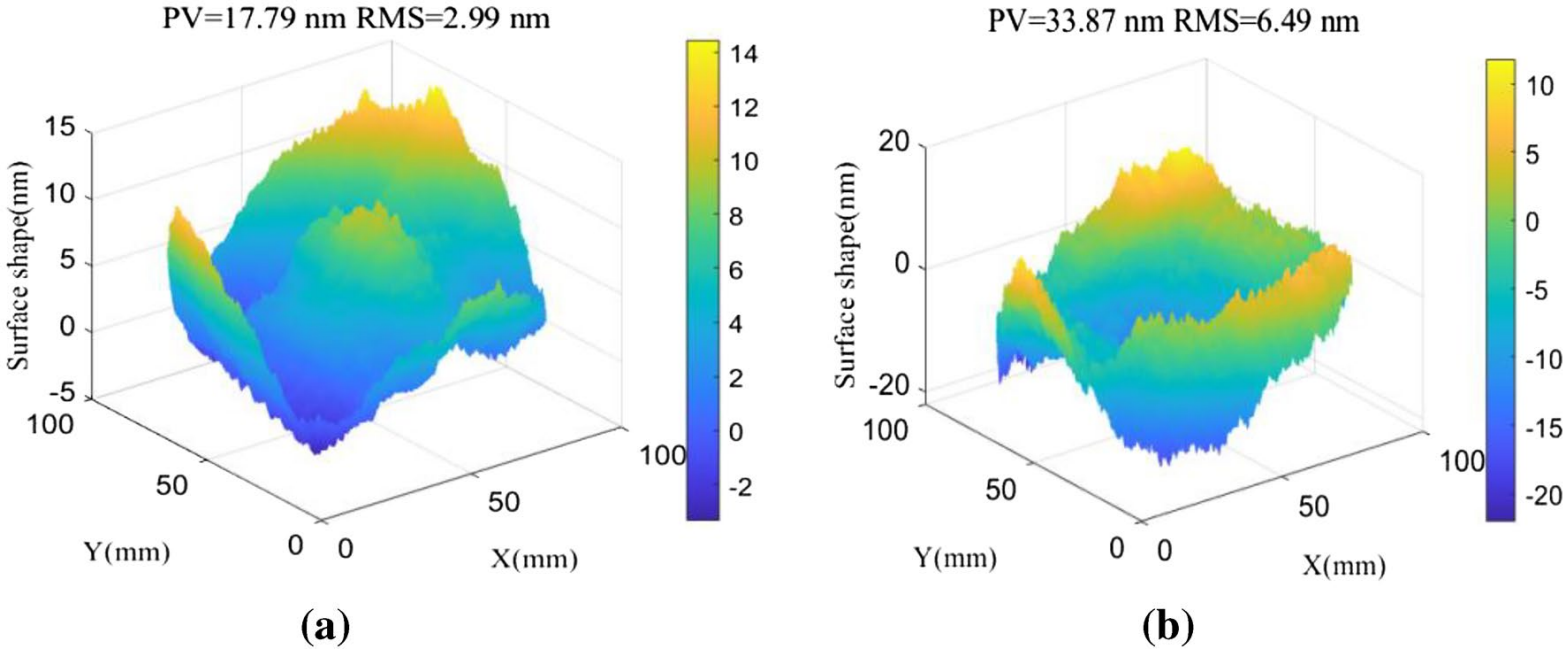
An error diagram for the initial middle and low frequency surface shapes. (**a**) The initial reference plane. (**b**) The initial measured surface.

The proposed conjugate double shift differential method was used to perform numerical calculations of surface shape reconstruction accuracy. Shape errors were first simulated for an initial reference surface and a measured surface. Low-frequency errors were generated using Zernike polynomial fitting, while intermediate-frequency errors were produced using a characteristic autocorrelation function. PV represents the difference between the maximum peak value and the minimum valley value of elements in the surface shape error matrix. RMS is the square root of the average of the square of a set of statistical data. It is a common indicator used to evaluate the surface shape of optical components, primarily for representing slow changes in the wave surface. As shown in Fig. 2, the PV value for the low-frequency shape error of the initial reference surface was 17.79 nm and the RMS value was 2.99 nm. The PV value for the initial measured surface was 33.87 nm and the RMS value was 6.49 nm. The simulation aperture was 100 mm in size and included a 500*500 grid of sampling points, with a corresponding resolution of 0.2 mm/pixel.

**Figure 3:**
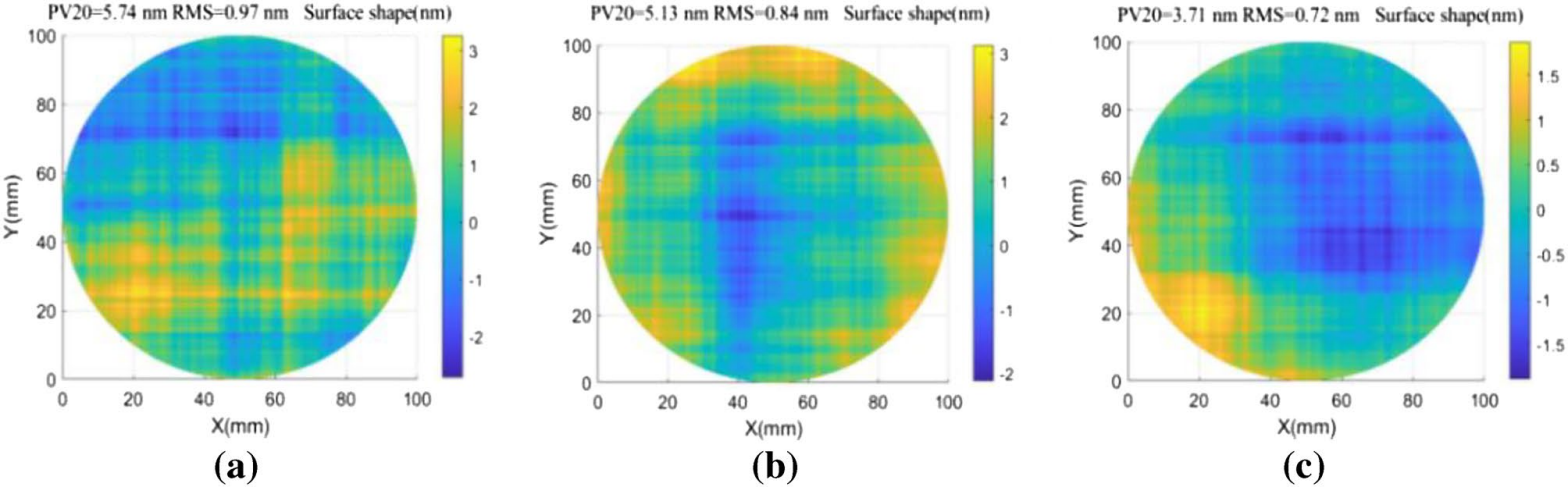
The residual shape of measured surfaces corresponding to different frequency spectrum compensation methods. (**a**) Direct zero filling. (**b**) Average neighboring points. (**c**) Conjugate double shift.

**Figure 4:**
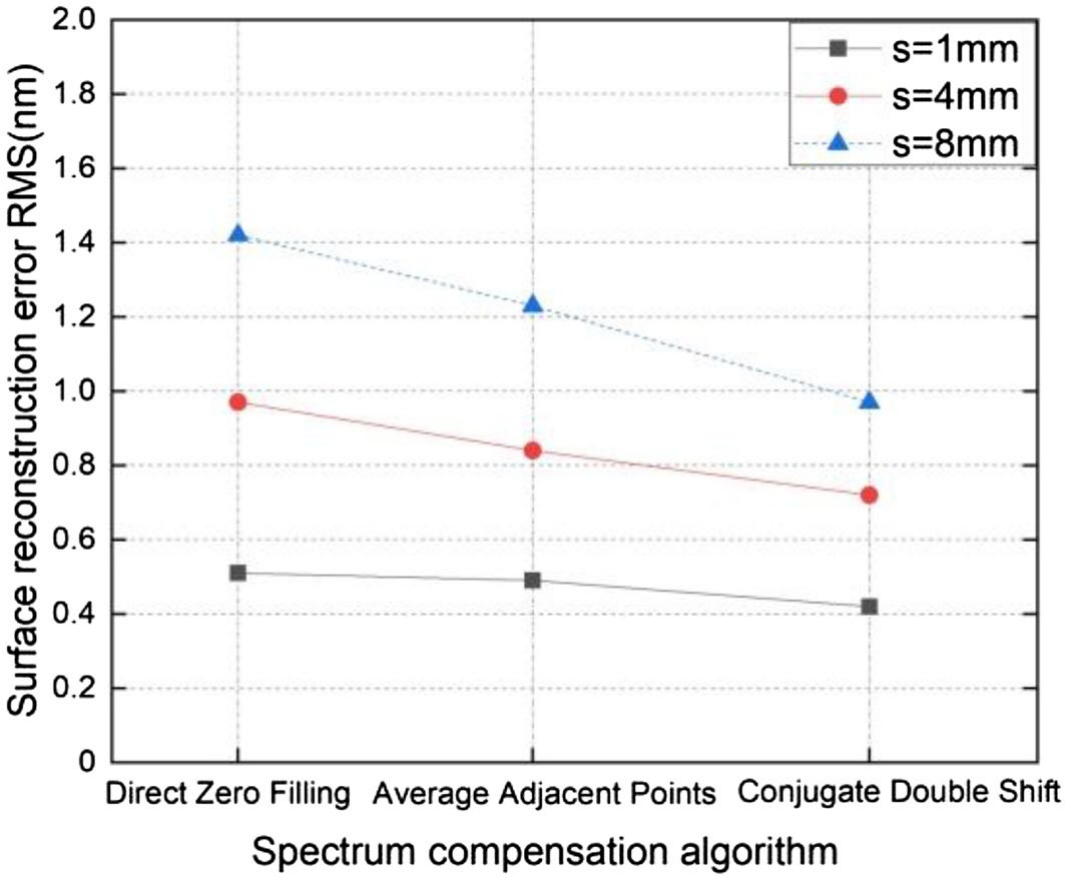
The influence of spectrum compensation on measurement results using various shift quantities.

Various spectrum compensation principles were used to reconstruct the Fourier transform wavefront on the simulation surface shown in Fig. 2. Since the Fourier algorithm can only process data in a rectangular domain, differential wavefronts in a circular domain were subject to continuation pretreatment prior to surface reconstruction. The two sets of shift quantities without a common factor were set as s_1_ = 4 mm(20 pix) and s_2_ = 4.2 mm(21 pix), while the residual shape of the measured surface was determined using direct zero filling. The average adjacent point and conjugate double shift methods are demonstrated in Fig. 3, where it is evident the residual error PV20 value produced by the direct zero compensation method was 5.74 nm and the RMS value was 0.97 nm. PV20 is the difference between the average of the top 10 points in the wave surface and the average of the bottom 10 points. This value can be used to reduce the influence of defects or remove outliers from the test data. The PV20 value acquired from adjacent points (in the averaging method) was 5.13 nm and the RMS value was 0.84 nm. The PV20 value produced by the conjugate double shift method was 3.71 nm and the RMS value was 0.72 nm. As such, the spectrum compensation accuracy produced by optimizing the shift method was superior to that achieved by interpolating existing data. The RMS values of surface reconstruction errors, calculated using different spectrum compensation methods, are shown for other shift quantities in Fig. 4.

A comparison of the three groups of broken lines in Fig. 4 indicates that larger shifts are indicative of more obvious spectrum loss and larger surface reconstruction errors. For a shift amount of 1 mm, the RMS value of the residual error was 0.51 nm for the zero-filling method, 0.49 nm for the averaging of adjacent points, and 0.42 nm for the conjugate double shift method (s_1_ = 1 mm and s_2_ = 1.2 mm). In other words, when the shift amount is small, less spectral information is lost and the resulting reconstruction accuracy of the conjugate shift differential method is equivalent for different spectrum compensation techniques. However, the conjugate double shift (s_1_ = 8 mm and s_2_ = 8.2 mm) algorithm also produced the highest accuracy for a shift value of 8 mm. In this case, the RMS value of the measurement residual error was reduced from 1.57 to 0.97 nm, which confirms the proposed technique can improve surface reconstructions by compensating for a lack of spectral response.

## Validation experiments

### Absolute testing of different spectrum compensation methods

**Figure 5:**
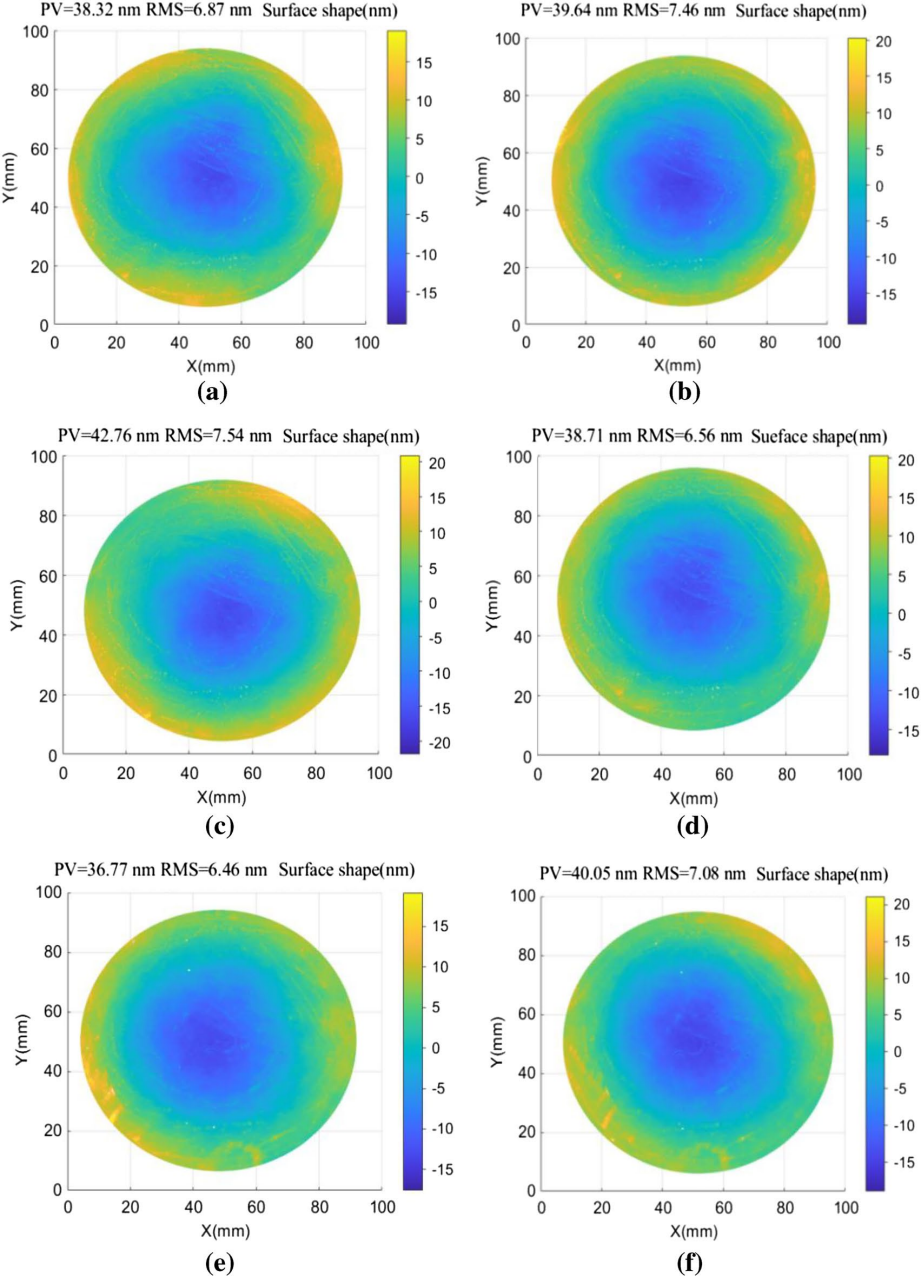

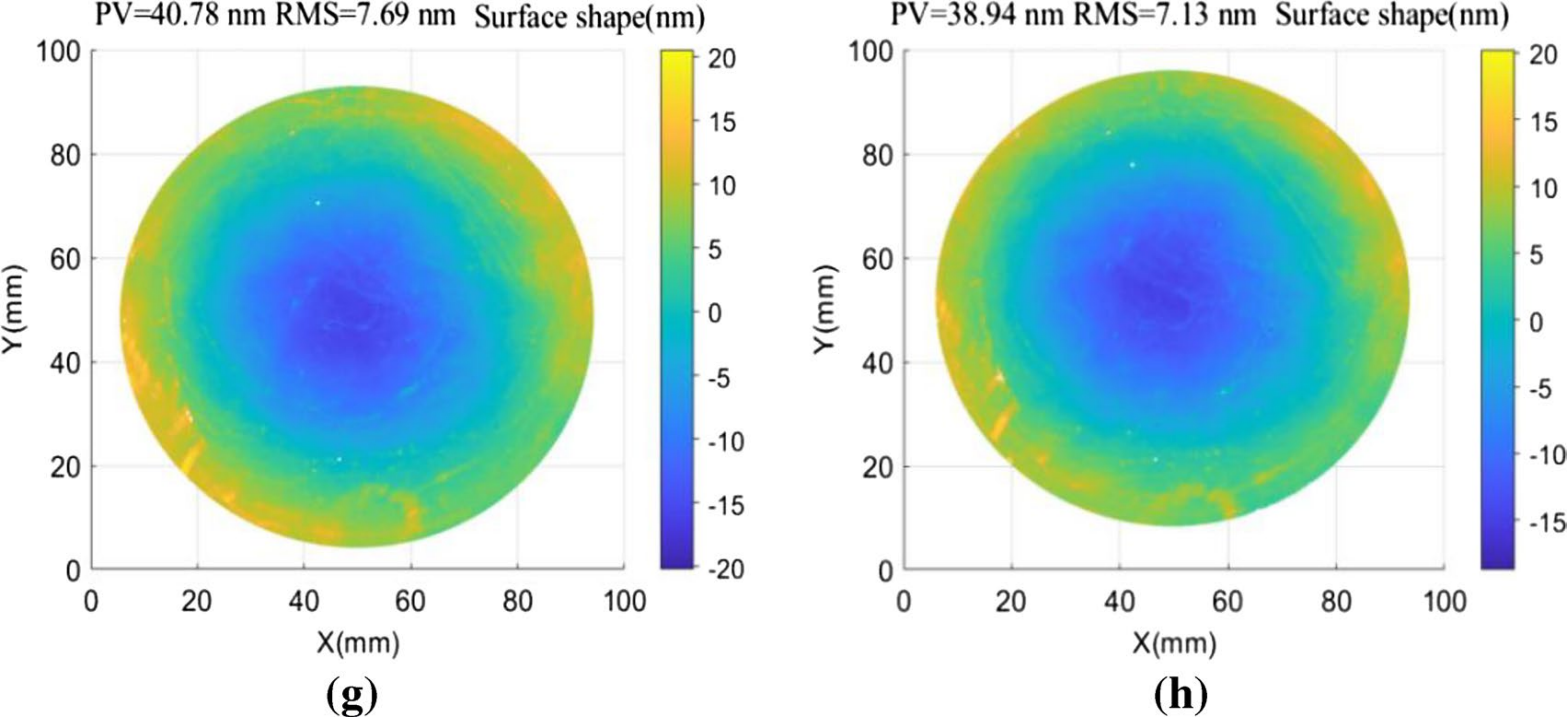
An interference wavefront diagram showing the resulting interference in various directions and at specific positions, including: (**a**) s_1_ = 4 mm in the − *x* direction, (**b**) s_1_ = 4 mm in the + *x* direction, (**c**) s_1_ = 4 mm in the − *y* direction, (**d**) s_1_ = 4 mm in the + *y* direction, (**e**) s_2_ = 4.3 mm in the − *x* direction, (**f**) s_2_ = 4.3 mm in the + *x* direction, (**g**) s_2_ = 4.3 mm in the − *y* direction, and (**h**) s_2_ = 4.3 mm in the + *y* direction.

**Figure 6:**
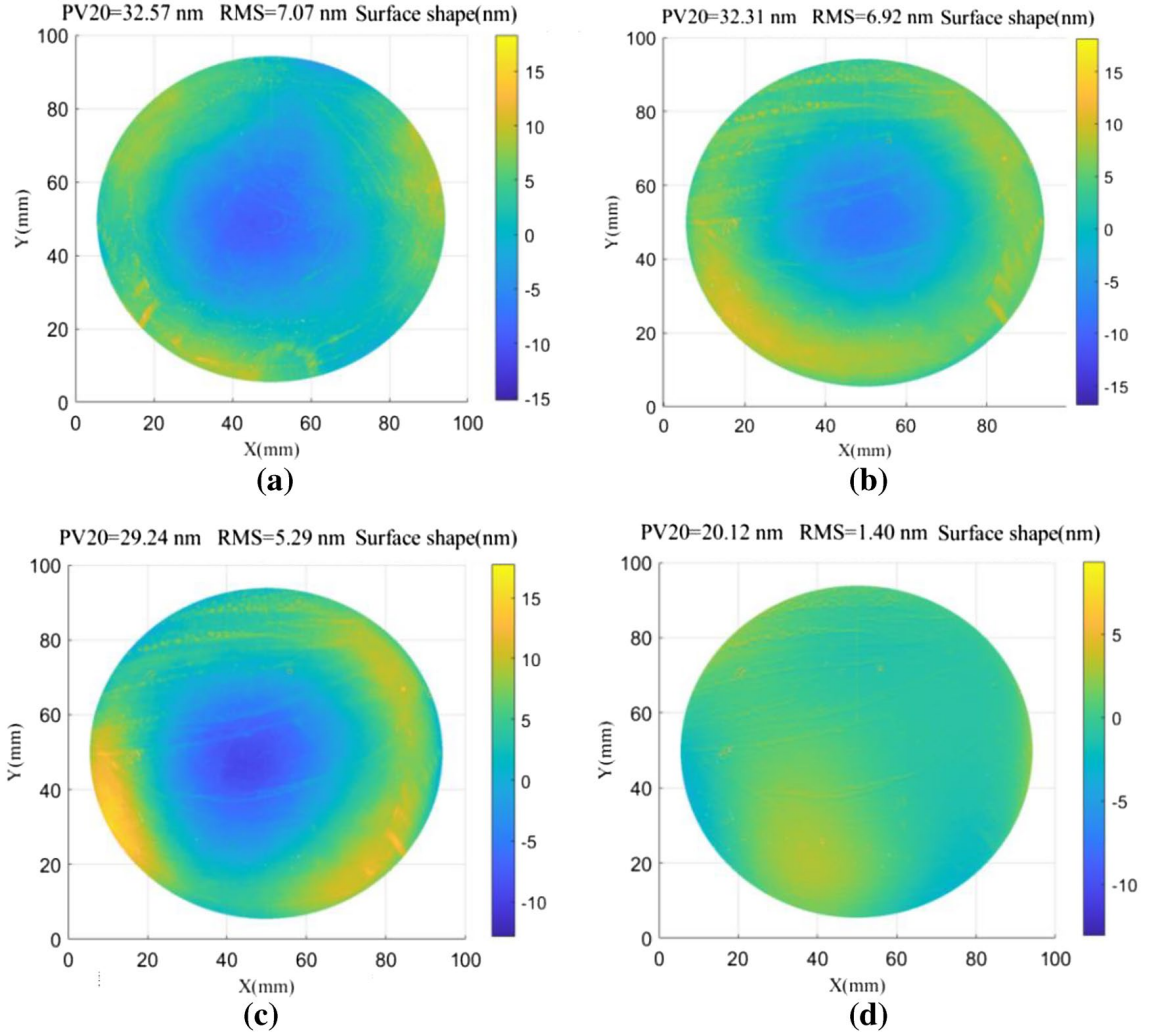
Wavefront reconstruction of measured surfaces using various spectrum compensation methods, including: (**a**) direct zero filling, (**b**) average neighboring points, and (**c**) conjugate double shift. (**d**) Surface shape deviations for the results shown in (**b** and **c**).

The effectiveness of the CDSD method in improving surface reconstruction accuracy was verified using a Zygo PE Fizeau phase-shifting interferometer with a high-precision displacement table. A plane standard mirror with a diameter of 100 mm was used in the experimental configuration, producing an interferometer pixel size of 0.1 mm/pix. Aperture effects were considered by removing 5% of the measured data at the edges. Noise was assumed to follow a random distribution with a mean value of *µ* = 0 and a standard deviation of *σ* = 2 nm. The PV value of the tested mirror was λ/40 and further analysis showed the optimal shift interval was 0.6–6.8 mm^17^. Figure 5 demonstrates a scenario in which shift values were s_1_ = 4 mm (40 pixels) and s_2_ = 4.3 mm (43 pixels). The test mirror was translated along the x and y directions of the reference mirror to obtain eight groups of wavefronts. Absolute surface shape testing based on zero filling and averaging of adjacent points was also performed for the interference wavefronts (a–d) shown in Fig. 5. Testing based on a conjugate double shift was performed for (a–h). Reconstruction results for the measured surface wavefronts are shown, along with measurement deviations, in Fig. 6.

### Results

**Figure 7:**
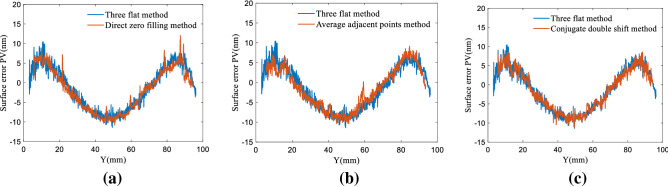
Cross-sectional line comparisons for the conjugate shift differential and three-sided mutual testing methods using different frequency spectrum compensation techniques, including: (**a**) direct zero filling, (**b**) average adjacent points, and (**c**) conjugate double shift.

The three-sided mutual inspection function module in the Zygo optical measurement software was used for comparative experimental analysis. Although three-sided mutual inspection can only acquire contour lines in a single direction, this is an ideal approach for comparative analysis of absolute surface testing because there is no principal error^18,19^. Figure 7 shows comparison results for the CSD and three-sided mutual inspection methods using different frequency spectrum compensation techniques. It can be seen from Fig. 7 that the CSD method based on Fourier transforms could reconstruct median and low-frequency shape errors for the measured surface. In Fig. 7a, the residual error (PV20 value) for the direct zero filling method is 11.35 nm and the RMS value is 1.72 nm. In Fig. 7b, the PV20 value for the average of adjacent points is 10.59 nm and the RMS value is 1.66 nm. In Fig. 7c, the PV20 value for the conjugate double shift method is 8.03 nm and the RMS value is 1.14 nm. Thus, the results of surface shape reconstruction using the proposed CDSD method are in good agreement with those of trihedral mutual testing, producing high measurement accuracy.

### Repeatability experiments

**Figure 8:**
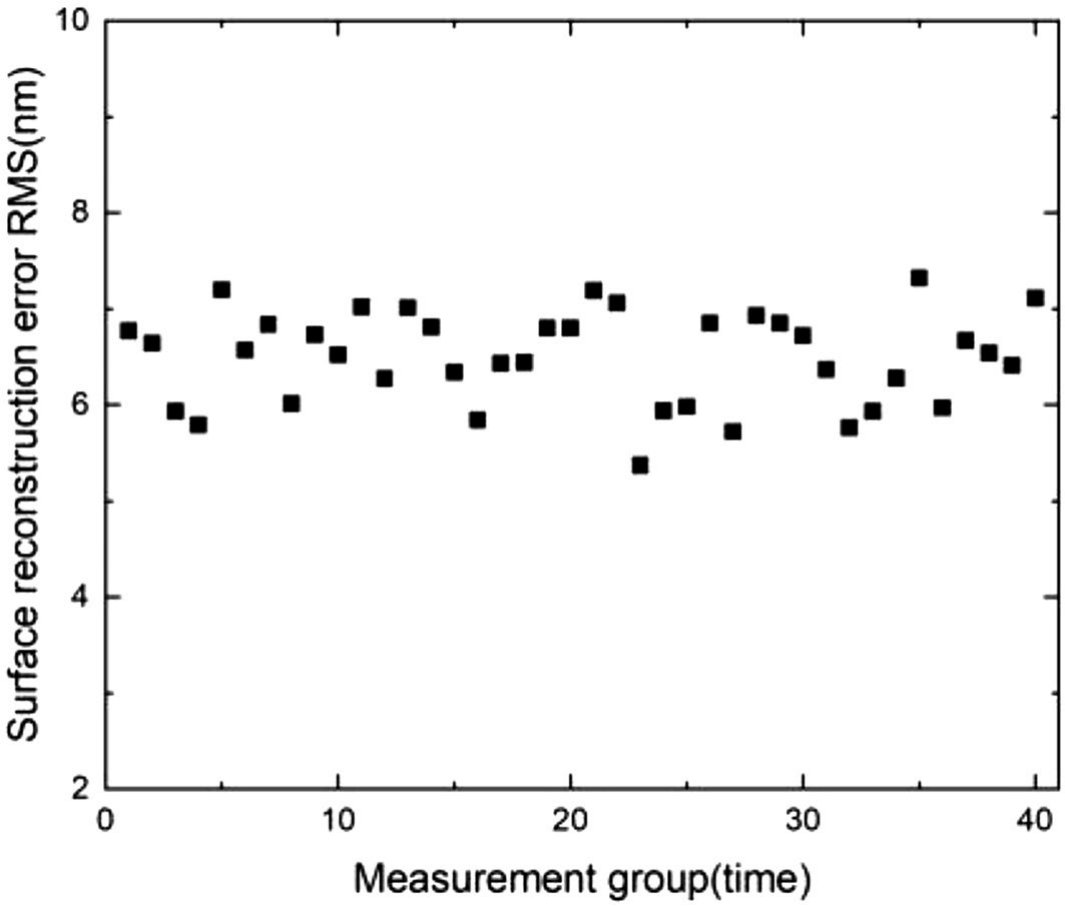
The results of absolute testing with conjugate double shift differential surface shapes for 40 samples.

The CDSD algorithm includes several measurement steps. As such, the measurement cycle is long and multiple uncertain factors are involved in the process, which can seriously affect algorithm stability. Therefore, measurement repeatability was evaluated using a series of experiments. The shift value s_1_ was 4 mm, while s_2_ was 4.3 mm across 40 sets of surface reconstruction errors, as shown in Fig. 8. Under the conditions of random noise and initial surface shape distributions, the optimal shift amount determined by the inverse optimization model can suppress the influence of random noise on measurement results^17^. In addition, the shift errors introduced by precision shift tables were small, so the repeatability of this approach is high. The RMS value for repeatability, calculated using Bessel’s formula, was 0.49 nm, which approaches that of commercial interferometers. These results suggest the proposed conjugate double shift differential method to be highly repeatable.

## Conclusion

This study combined conjugate shift differential absolute testing technology with the double shear effect in transverse shear interferometry, thereby introducing a novel conjugate double shift differential (CDSD) method for accurate measurement of optical element surfaces. This technique can reduce the influence of missing spectra on measurement accuracy in Fourier wavefront reconstructions, particularly for high-precision absolute testing of optical elements. When no common factors exist between two groups of shift quantities, spectrum information lost in the first group of shape measurements can be compensated for using the second group of experimental data. Accurately measured profiles can then be obtained by an inverse Fourier transform of the wave surface coefficients after spectrum compensation. Experimental results for planar optical elements using different spectrum compensation methods showed that, compared with direct zero compensation and adjacent point averaging, the RMS value of the measurement error for the CDSD method was reduced from 1.72 to 1.14 nm, with a repeatability of 0.49 nm. This verifies the effectiveness and repeatability of the proposed algorithm in compensating for missing spectra and improving the accuracy of wavefront reconstructions. This work could further improve surface measurements for planar optical elements, thereby promoting the wide applicability of Fourier reconstruction algorithms in absolute testing technology.

## Data Availability

The datasets used and analyzed during the current study are available from the corresponding author on reasonable request.
